# Expression and cilia associated localization of Histone deacetylases 6 in *Xenopus*

**DOI:** 10.17912/micropub.biology.000919

**Published:** 2023-08-14

**Authors:** Matthias Tisler, Tim Ott, Martin Blum, Axel Schweickert

**Affiliations:** 1 Department of Zoology, University of Hohenheim, Stuttgart, Baden-Württemberg, Germany; 2 Institute of Diagnostic and Interventional Radiology, Technical University of Munich, Munich, Bavaria, Germany

## Abstract

Histone deacetylases (HDACs) are key posttranslational modulators of the proteome. We show that expression of *histone deacetylase 6* ( *hdac6* ) is dynamic and appears in a tissue specific manner throughout embryonic development of the frog Xenopus *laevis* . Interestingly, *hdac6* transcripts often associate with ciliated tissues, like the left-right organizer at neurula stage or the pronephros. In the embryonic skin, Hdac6 protein localizes to the cilia base, suggesting a functional link.

**Figure 1. Expression of Xenopus hdac6 on mRNA and protein level:**
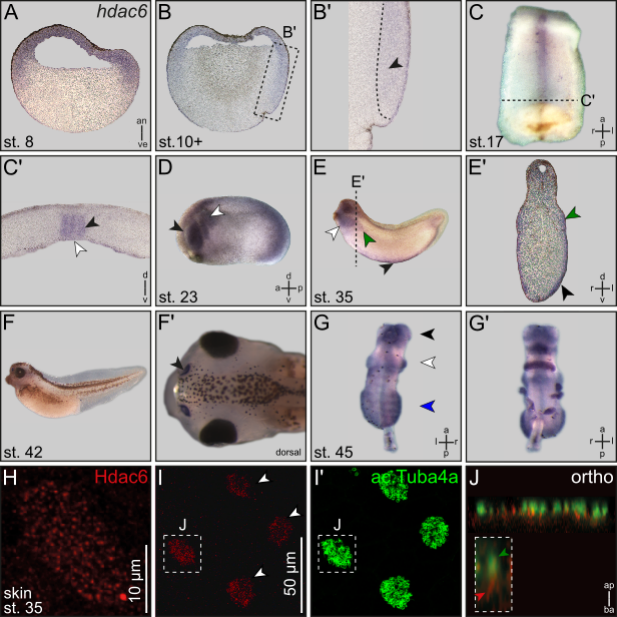
(A) Sagital section of a blastula stage *Xenopus* embryo. *hdac6* is ubiquitously expressed in cells that form the animal hemisphere of the frog embryo. (B) Sagittal section of a gastrula embryo. *hdac6* signal is detectable in the ectoderm, cells of the superficial mesoderm and cells of mesodermal fate (black arrow head in magnification B’). (C) Ventral view of a dorsal explant of a neurula embryo. (C’) Transversal section of C. *hdac6* expression is restricted to the notochord and cells of the gastrocoel roof plate i.e. the LRO (black and white arrow head, respectively). (D) *hdac6* is expressed in the developing eye cup (black arrow head) and in migrating neural crest cells (white arrow head). (E) *hdac6* transcripts appear in the pronephric kidney (green arrow head), ventral hematopoietic stem cells (black arrow head) and in the developing craniofacial cartilage (white arrow head). (E’) transversal section of E. (F) *hdac6* activity in larval nasal pits. (F’) Dorsal view of tadpole shown in F. Nasal pit is highlighted (black arrow head). (G) Dorsal view of pre-metamorphic brain explant. *hdac6* shows a regionalized expression pattern in the tel-, di- and rhombencephalon (black, white or blue arrowheads, respectively). (G’) Ventral view of the brain explant shown in G’. (H) Immunofluorescent staining of Hdac6 (red) in the larval epidermis at stage 35. Hdac6 shows a spotted expression pattern within epidermal cells. Staining of acetylated alpha-Tubulin (ac.Tuba4a) by immunofluorescence (green) reveal that Hdac6 positive cells are multiciliated cells (MCCs). J in I and I’ indicates MCC shown in J. (J) Orthogonal section of a MCC stained against Hdac6 (red) and ac.Tuba4a (green). Hdac6 localizes at the base of motile cilia. Inserted box shows a magnification of a single cilium. The ciliary axonem is highlighted by a green arrow head, Hdac6 localization is indicated by a red arrow head. Scale bar: 10µm (I) Hdac6 is detected in a distinct subtype of epidermal cells (white arrowheads) Scale bar: 50µm. (I’)

## Description

Histone deacetylases (HDACs) are best known for their transcriptional regulation in eukaryotic cells, by controlling DNA accessibility for e.g. transcription factors via deacetylation of histone proteins (Seto and Yoshida, 2014) . In higher vertebrates HDACs form an evolutionary conserved but diverse family of 11 genes that can be grouped into four subfamilies (class I to IV; Milazzo et al., 2020).These enzymes localize to the nucleus and / or the cytoplasm, allowing specific interaction with distinct molecular substrates. By deacetylating lysine residues of histone or non-histone proteins, HDACs play a key role in the post-translational modification of proteins, mediating general transcriptional activation or modulation of signaling pathways (Zhu et al., 2023) .

The highly conserved and best analyzed HDAC6 (subclass IIb) predominantly localizes to the cytoplasm and was shown to serve as the major deacetylase of acetylated alpha-Tubulin (Hubbert et al., 2002) . The stand-alone feature of HDAC6 is the functional duplication of its deacetylase domain. Interestingly, each domain appears to have a distinct set of molecular substrates (Milazzo et al., 2020) . Moreover, HDAC6 has been identified as a reasonable player in the pathophysiological process of different types of cancer and serves therefore as a potentially druggable therapeutic target protein (Li et al., 2018) .

Surprisingly, *Hdac6* knockout mice are viable and fertile (Haberland et al., 2009; Zhang et al., 2008) . Although lacking an obvious developmental phenotype, HDAC6 depleted mice show defects in bone mineralization and alterations regarding the immune response and emotional behavior (Fukada et al., 2012; Zhang et al., 2008) . Experiments in Hdac6 depleted zebrafish strains also point towards a subtle but tissue specific loss-of-function (LOF) phenotype, affecting organs like brain, eye, inner ear and kidney leading to variations in movement behavior (Łysyganicz et al., 2021). To date, little is known about an early embryonic function of Hdac6 in *Xenopus* , with the exception of its contribution to the development of the visual system in swimming tadpoles (Bestman et al., 2015) . Here, we report the tissue specific developmental expression pattern of *hdac6* mRNA in combination with its protein localization, which indicate an association with cilia.

In order to analyze the expression pattern of *hdac6* throughout *Xenopus* development, we performed whole-mount mRNA *in* *situ* hybridization experiments. *hdac6* transcripts are ubiquitously detectable in the animal hemisphere of stage 8 blastula embryos ( Fig. 1A ). During gastrulation, expression is found in the ectoderm ( Fig. 1B ) as well as in dorsal mesodermal cells including the superficial mesoderm ( Fig. 1B ’, Shook et al., 2004), which will later form the ciliated left-right organizer (LRO, Schweickert et al., 2007). In dorsal explants of stage 17 neurulas, *hdac6* signal is restricted to the cells of the axial mesoderm, i.e. notochord and the epithelial layer of the gastrocoel roof plate, harboring the LRO ( Fig. 1C, C ’). Vibratome sections of stage 17 dorsal explants reveal that *hdac6* expression is detectable in fluid flow generating cells of the LRO ( Fig. 1C ’, Schweickert et al., 2007; Blum et al., 2009). Stage 23 tailbud embryos express *hdac6* in migrating neural crest cells as well as in the developing eye cup ( Fig. 1D ). Later in development, around stage 30, additional expression domains get established in the developing pronephros and in the ventral hematopoietic stem cell niche ( Fig. 1E, E ’). At stage 42, *hdac6* expression is exclusively detectable in nasal pits of the tadpole ( Fig. 1F, F ’). Whole brain explants show a distinct and regionalized *hdac6* expression in the larval brain at stage 45 in the telencephalon, diencephalon and rhombencephalon ( Fig. 1 G, G ’).

The ectoderm, the superficial mesoderm and the forming pronephros eventually mature into tissues with motile and / or non-motile cilia. In a next step, we wanted to investigate a potential cilia associated localization of Hdac6 protein via immunofluorescence (IF). Short of antibodies directed against *Xenopus* Hdac6, we used a commercially available polyclonal antibody against human HDAC6. Although, homology of human and frog sequences is high, antibody cross-reactivity to unrelated *Xenopus* proteins cannot be excluded. The *Xenopus* epidermis is an exemplary mucociliary epithelium and ideal model system for ciliary protein localization studies (Walentek, 2021) . In stage 35 embryos, Hdac6 was found to localize in a punctate pattern to a specific subset of epidermal cells ( Fig. 1H, I ). A combined IF staining against acetylated alpha-Tubulin revealed Hdac6 localization in multiciliated cells (MCCs) of the skin ( Fig. 1I ’). Orthogonal sections further show that Hdac6 is enriched at the cilia base ( Fig. 1J ). Although being characterized as an important tubulin deacetylase *in vivo* (Hubbert et al., 2002; Zhang et al., 2003) Hdac6 is not detected in the ciliary axoneme ( Fig. 1J ).

Published data have implicated a role of Hdac6 in cilia homeostasis of different vertebrate species (Łysyganicz et al., 2021; Yang et al., 2014). The tissue specific developmental mRNA expression pattern in *Xenopus* is in line with the previously reported, subtle LOF phenotypes in the corresponding embryonic tissues (Bestman et al., 2015; Łysyganicz et al., 2021; Zhang et al., 2008). Additionally, the observed localization of Hdac6 protein at the base of motile cilia points towards a more complex role of Hdac6 rather than exclusively regulating the axonemal acetylation of alpha-Tubulin. *hdac6* expression in the LRO of the frog embryo argues for a functional contribution of Hdac6 in left-right patterning. Indeed, chemical modifier screens have previously identified HDAC inhibitors like Trichostatin A or Sodium butyrate as left-right defects causing agents whereas the mode of action remains elusive (Cao et al., 2009; Carneiro et al., 2011) .

As *Xenopus* serves as an ideal model for the investigation of ciliary architecture and function in health and disease (Blum and Ott, 2018) , future experiments might elucidate the exact role of *histone deacetylase 6* in context of mucociliary epithelia and its functional role in the establishment of visceral left-right asymmetry.

## Methods

Materials and methods

*Animal care and maintenance*

Husbandry, handling and experimental manipulations of *Xenopus laevis* frogs/embryos were approved by the Regional Government Stuttgart, Germany (A379/12 Zo, ‘Molekulare Embryologie’, V340/17 ZO and V349/18 ZO, ‘ *Xenopus* Embryonen in der Forschung’) and performed according to German federal laws and regulations (§6, article 1, sentence 2 nr. 4 of the animal protection act). Animals were staged according to (Nieuwkoop and Faber, 1994) .

*Xenopus laevis hdac6*

A full ORF clone from *Xenopus laevis hdac6* cDNA (Ref seq; NM_001087017 Unigene ID Xl.8310) in pCMV SPORT6 (clone IRBHp990G107D) vector was purchased from Source BioScience (Cambridge, UK) and linearized with *NcoI* for antisense transcription with T7 polymerase (Promega).

*RNA in situ hybridization and histological analysis*

Embryos dedicated for *in situ* hybridization were fixed in MEMFA for 2 h at RT and later on processed following standard protocols suitable for *Xenopus* embryos (Sive et al. 2007) . Digoxigenin-labled (Roche) antisense-RNA probes were generated via *Sp6* or *T7* RNA Polymerase (Promega) from linearized plasmids. mRNA-ISH was performed according to (Belo et al., 1997) . For further histological analysis embryos were embedded in gelatine–albumin and sectioned (35 μm) on a Leica VT1000S vibratome.

*Immunofluorescence*

Endogenous *Xenopus laevis* Hdac6 Protein was detected by a commercially available antibody (Sigma-Aldrich; SAB1406911; 1:250) after overnight fixation (-20 °C) using Dent's fixative (80 % methanol; 20 % DMSO). Cilia located at the tadpole epidermis were visualized as described by Vick et al. 2009 After fixation embryos were treated according to standard IF procedures (Sive et al. 2007) . Antibodies used were: mouse monoclonal antibody directed against acetylated alpha-Tubulin (Sigma-Aldrich; T6793; 1:700) and Alexa488 or Cy3-conjugated secondary polyclonal anti-mouse antibodies (Invitrogen; A11059 or Sigma-Aldrich; C2181; both 1:250).
